# Genome sequences of 36 *Streptococcus pneumoniae* strains optimized for the multiplexed opsonophagocytosis killing assay

**DOI:** 10.1128/mra.00553-24

**Published:** 2024-08-20

**Authors:** Emma Graffice, Chloe Meewes, Feroze A. Ganaie, Moon H. Nahm, Juan J. Calix

**Affiliations:** ^1^Department of Medicine, Division of Infectious Disease, University of Alabama at Birmingham, Birmingham, Alabama, USA; 2 Division of Pulmonary/Allergy/Critical Care, Department of Medicine, University of Alabama at Birmingham, Birmingham, Alabama, USA; DOE Joint Genome Institute, Berkeley, California, USA

**Keywords:** *Streptococcus pneumoniae*, MOPA, WGS

## Abstract

A multiplexed opsonophagocytosis assay (MOPA) was developed as a cost-effective, high-throughput biological assay to evaluate the efficacy of pneumococcal vaccines by *in vitro* measurement of opsonophagocytic activity of anti-capsular antibodies. Here, we report draft genomes of the 36 strains of *Streptococcus pneumoniae* developed for use in the reference pneumococcal MOPA.

## ANNOUNCEMENT

*Streptococcus pneumoniae* (the pneumococcus) is a human pathobiont that expresses one of >100 capsule polysaccharides synthesized by genes located in its capsule synthesis (*cps*) locus (1). Antibodies targeting capsule can mediate type-specific protection via complement-dependent opsonophagocytic killing, and vaccines that target up to 23 of the prevalent capsule types (serotypes) have reduced the global burden of pneumococcal disease. The pneumococcal multiplexed opsonophagocytosis assay (MOPA) is a tool that directly measures antibody-mediated killing of bacteria and is used to validate vaccine efficacy (2, 3). Since the assay’s development in 2000 (4), the number of MOPA strains has expanded to incorporate serotypes used in subsequent vaccine formulations (5–7). We sequenced the genomes of all 36 *S*. *pneumoniae* strains currently employed in MOPA protocols to increase their utility in ongoing research.

Strains were obtained from the Nahm lab and can be acquired from BEI Resources (Table 1) or upon request from UAB. Isolates were grown overnight at 37°C with 5% CO_2_ on sheep blood agar. DNA was extracted using the NEB Monarch Genomic DNA libraries were prepared with Illumina DNA Prep Kit and IDT 10 bp UDI, and sequenced with Illumina NextSeq 2000 at SeqCenter (Pittsburgh, PA). An average of 3.5 million 2 × 151 bp reads was obtained per genome. Demultiplexing, quality control, and adapter trimming were performed with bcl-convert (v3.9.3) (Illumina, San Diego, CA). Genomic raw reads were assembled *de novo* using Unicycler (v0.5.0) (8), assembly statistics were assessed using Quast (v5.2.0) (9), and coding regions were identified with NCBI Prokaryotic Genome Annotation Pipeline (10). PyANI (v0.2.12) (11, 12) confirmed genomes shared ≥96% average nucleotide identity with NCBI pneumococcal reference genome (GCF_002076835.1). Completeness and contamination were determined using CheckM (v1.2.2) (13). Multilocus sequence type (MLST) was determined using MLST (v2.23.0) (14) and the PubMLST database for *S. pneumoniae* (15). Molecular *cps* typing was done by PneumoKITy (v1.2.1) (16), and *cps* identity was confirmed by aligning gene nucleotides and encoded amino acid sequence to references as listed in Table 1 (17–25). All tools were used with default parameters unless noted otherwise. Confirmatory serotyping was done as described previously with multibead assay (26), flow cytometry serotyping assay (27), quellung (28), and agglutination, using polyclonal rabbit antisera and in-house monoclonal antibodies (26, 29). Antimicrobial selection for each isolate was previously published in the MOPA protocol (2, 3).

**TABLE 1:** Description of accession numbers and genomic metadata

					NCBI accession numbers			Genome characteristics									PneumoKITy *cps* locus typing		Manual *cps* locus typing			
MOPA strain	BEIcatalog#^*a*^	Parentstrain	Serotype^*b*^	Antibioticresistance	BioSample	SRA	Assembly	Length (bp)	%GC	Contigs (*n*)	N50	Estimated depth	CDS^*d*^	Completeness (%)	Contamination (%)	MLST profile^*e*^	*cps*locustypecall	%Nucleicacididentity	*cps*locustype name^*c*^	Reference number	Reference accession	%Nucleicacididentityto reference
EMC23F	NR-51859	1212458	23F	Trimethoprim	SAMN38501028	SRR26988276	JAXIJT000000000	2,030,044	39.62	66	101,509	129.4	2,101	99	0.31	507	23F	98.6%	“23F”	24	CR931685.1	99.84
EMC9V	NR-51855	1081748	9V	Streptomycin	SAMN38501029	SRR26988275	JAXIJS000000000	2,071,893	39.58	57	89,409	108.7	2,124	99.12	0.62	162	9A/9V	98.6%	“9V”	24	CR931648.1	99.98
OREP10A	NR-59133	DS3032-06	10A	Optochin	SAMN38501030	SRR26988264	JAXIJR000000000	2,046,505	39.53	60	113,563	106.0	2,083	99.02	0.33	816	10A	98.6%	“10A”	24	CR931649.1	99.29
OREP17F	NR-59136	DS3022-06	17F	Optochin	SAMN38501031	SRR26988253	JAXIJQ000000000	2,067,337	39.5	65	87,167	108.0	2,109	99.04	0.52	13,918	17F	98.6%	“17F”	24	CR931670.1	99.99
OREP18C	NR-51856	GP116	18C	Optochin	SAMN38501032	SRR26988246	JAXIJP000000000	2,097,585	39.64	90	83,584	102.6	2,200	99.12	0.36	4,706	18C/18B	98.6%	“18C”	24	CR931673.1	100
OREP23B		DS4792-06	23B	Optochin	SAMN38501033	SRR26988245	JAXIJO000000000	2,119,987	39.46	49	136,097	91.7	2,145	99.16	0.31	4,507	23B	98.6%	“23B1”	23	LT594598.1	99.83
OREP3	NR-51850	WU2	3	Optochin	SAMN38501034	SRR26988244	JAXIJN000000000	1,964,466	39.71	55	89,179	302.2	2,011	99.16	0.31	378	3	98.6%	“3”	24	CR931634.2	99.72
OREP4	NR-51851	DS2382-94	4	Optochin	SAMN38501035	SRR26988243	JAXIJM000000000	2,070,881	39.53	93	69,622	145.1	2,111	98.96	0.62	NT^*f*^	4	98.6%	“4”	24	CR931635.1	99.98
OREP7F	NR-51854	DS2617-97	7F	Optochin	SAMN38501036	SRR26988242	JAXIJL000000000	1,973,977	39.8	76	69,278	112.3	2,012	98.12	0.31	191	7F/7A	98.6%	“7F”	24	CR931643.1	99.85
SPEC1	NR-51849	L82006	1	Spectinomycin	SAMN38501037	SRR26988241	JAXIJK000000000	2,075,254	39.53	75	74,828	117.9	2,146	99.27	0.31	227	1	98.5%	“1”	24	CR931632.1	99.98
SPEC15C		DS3031-06	15C	Spectinomycin	SAMN38501038	SRR26988274	JAXIJJ000000000	2,050,685	39.55	48	120,019	117.4	2,051	99.3	0.47	199	15B/15C	98.7%	“15C”	24	CR931665.1	99.35
SPEC19F	NR-51858	DS2217-94	19F	Spectinomycin	SAMN38501039	SRR26988273	JAXIJI000000000	2,109,586	39.53	89	69,211	88.4	2,157	99.13	0.62	7,221	19F	98.6%	“19F”	24	CR931678.1	99.26
SPEC20A		6320	20A	Spectinomycin	SAMN38501040	SRR26988272	JAXIJH000000000	2,080,592	39.53	49	128,663	112.4	2,126	99.27	1.46	1,257	20	98.6%	“20A”	18	JQ653094.1	99.99
SPEC20B	NR-59137	CDC3014-06	20B	Spectinomycin	SAMN38501041	SRR26988271	JAXIJG000000000	2,029,478	39.54	53	112,364	126.2	2,060	99.22	1.25	10,479	20	98.7%	“20B”	18	JQ653093.1	99.99
SPEC31		DS3159-06	31	Spectinomycin	SAMN38501042	SRR26988270	JAXIJF000000000	1,986,319	39.7	55	83,427	123.6	2,016	98.78	0.62	452	31	98.6%	“31”	24	CR931695.1	99.98
SPEC35B		DS3094-06	35B	Spectinomycin	SAMN38501043	SRR26988269	JAXIJE000000000	2,082,372	39.57	76	82,677	104.5	2,150	98.52	0.31	393	35B/35D	98.6%	“35B”	24	CR931705.1	100
SPEC38		DS3025-06	38	Spectinomycin	SAMN38501044	SRR26988268	JAXIJD000000000	2,086,548	39.5	90	60,367	83.8	2,125	99.02	0.62	393	38	98.6%	“38”	24	CR931710.1	99.98
SPEC6B	NR-51853	BG25-9	6B	Spectinomycin	SAMN38501045	SRR26988267	JAXIJC000000000	2,068,259	39.69	80	74,304	113.2	2,116	99.12	0.31	385	6E	98.6%	“6E”	19	AF246897.1	99.93
SPEC6C	NR-20805	BGO-2197	6C	Spectinomycin	SAMN38501046	SRR26988266	JAXIJB000000000	2,036,461	39.65	47	125,656	119.7	2,090	99.27	0.31	2,899	6C/6D	98.6%	“6C”	20	JF911515.1	99.48
SPEC6D	NR-20806	MNZ920	6D	Spectinomycin	SAMN38501047	SRR26988265	JAXIJA000000000	2,045,865	39.61	45	143,727	133.1	2,068	98.89	0.31	5,163	6A/6B/6C/6D	98.6%	“6D”	21	HM448897.1	99.97
SPEC9N	NR-31702	CDC1398-00	9N	Spectinomycin	SAMN38501048	SRR26988263	JAXIIZ000000000	2,062,705	39.53	44	118,156	125.2	2,062	99.12	0.31	66	9N/9L	98.6%	“9N”	24	CR931647.1	100
STREP14		DS2214-94	14	Streptomycin	SAMN38501050	SRR26988261	JAXIIX000000000	2,072,127	39.68	86	94,578	137.1	2,170	99.21	0.31	124	14	98.6%	“14”	24	CR931662.1	99.94
STREP2	NR-53529	D39	2	Streptomycin	SAMN38501051	SRR26988260	JAXIIW000000000	2,007,772	39.67	59	74,135	125.6	2,038	98.97	0.31	595	2	98.7%	“2”	24	CR931633.1	99.98
STREP23A		DS4063-06	23A	Streptomycin	SAMN38501052	SRR26988259	JAXIIV000000000	2,083,834	39.59	53	130,135	137.5	2,149	98.87	0.38	338	23A	99.0%	“23A”	24	CR931683.1	99.72
STREP24F		MNK0474	24F	Streptomycin	SAMN38501053	SRR26988258	JAXIIU000000000	2,230,581	39.39	76	124,071	114.9	2,312	99.35	0.57	3,386	24F/24B	98.5%	“24C”	22	MW683300.1	99.99
STREP33F		DS3052-06	33F	Streptomycin	SAMN38501054	SRR26988257	JAXIIT000000000	2,053,257	39.59	36	90,893	274.0	2,110	98.82	0.31	2,705	33F/33A	98.6%	“33F”	24	CR931702.1	99.98
STREP5		DBL5	5	Streptomycin	SAMN38501055	SRR26988256	JAXIIS000000000	2,026,268	39.59	34	171,835	110.0	2,058	98.34	0.38	4,840	5	98.6%	“5”	24	CR931637.1	100
STREP8		CDC5675-06	8	Streptomycin	SAMN38501056	SRR26988255	JAXIIR000000000	1,976,195	39.7	51	148,970	318.1	2,031	98.98	0.64	1,480	8	98.6%	“8”	24	CR931644.1	99.98
TREP11A		CDC3160-06	11A	Trimethoprim	SAMN38501057	SRR26988254	JAXIIQ000000000	1,988,348	39.7	47	143,880	241.1	2,025	99.12	0.31	62	11A/11D	98.7%	“11A-1”	17	GU074952.1	99.97
TREP12F	NR-59134	DS4009-06	12F	Trimethoprim	SAMN38501049	SRR26988262	JAXIIY000000000	1,969,176	39.76	45	125,658	125.6	1,990	99.23	0.31	220	12F	98.6%	“12F”	24	CR931660.1	99.7
TREP15A		DS3148-06	15A	Trimethoprim	SAMN38501058	SRR26988252	JAXIIP000000000	2,038,709	39.51	60	101,093	128.9	2,068	98.65	0.33	63	15A	98.6%	“15A”	24	CR931663.1	99.75
TREP15B	NR-59139	CDC0556-97	15B	Trimethoprim	SAMN38501059	SRR26988251	JAXIIO000000000	2,051,575	39.54	44	141,424	128.8	2,051	99.35	0.47	199	15B/15C	98.7%	“15B”	24	CR931664.1	99.38
TREP16F		DS3042-06	16F	Trimethoprim	SAMN38501060	SRR26988250	JAXIIN000000000	2,032,670	39.63	64	105,496	129.4	2,083	98.91	0.31	659	16F	98.6%	“16F”	24	CR931668.1	99.98
TREP19A	NR-51857	DS3519-97	19A	Trimethoprim	SAMN38501061	SRR26988249	JAXIIM000000000	2,042,960	39.56	63	91,999	113.2	2,077	98.8	0.47	199	19A	98.6%	“19A”	24	CR931675.1	99.99
TREP22F		DS3433-06	22F	Trimethoprim	SAMN38501062	SRR26988248	JAXIIL000000000	2,051,979	39.55	56	95,952	123.5	2,113	99.32	0.68	433	22F	98.6%	“SSI-22F”	23	LT594600.1	99.98
TREP6A	NR-51852	EF6796	6A	Trimethoprim	SAMN38501063	SRR26988247	JAXIIK000000000	2,117,127	39.53	62	103,267	298.1	2,166	98.83	0.7	460	6A/6B	98.6%	“6A”	24	CR931638.1	99.99

^*a*^ If not listed, the strains can be made available upon request to the Nahm Lab.

^*b*^ Serotype was determined by combination of assays: multibead assay, flow cytometry-based serotyping assay, quellung, and agglutination using polyclonal rabbit antisera and in-house monoclonal antibodies.

^*c*^ *cps* locus types according to the references (listed in "Reference number" column) in which they were first linked to expression of their respective serotypes.

^*d*^ CDS is the number of coding sequence regions.

^*e*^ MLST = multilocus sequence typing.

^*f*^ NT describes non-typeable multilocus sequence type.

The *cps* type of previously described MOPA strains (22, 25) was confirmed, and we identified the closest related *cps* type of each other strain (Table 1). Notably, TREP22F contained the SSI-22F *cps* locus type first described by Kapatai et al. (23). *wcwC* found in the reference 22A and 22F *cps* loci reported by Bentley et al. (24) is replaced by an unrelated glycosyltransferase in SSI-22F, supporting that this *cps* gene determines structural differences between 22A and 22F. Thus, molecular typing strategies should use the SSI-22F *cps* locus sequence as the 22F reference sequence, as previously proposed (23). Altogether, MOPA strains represent a diversity of pneumococcal lineages and contain *cps* types that are concordant with their expressed serotypes.

## Data Availability

All data are available on NCBI in BioProject PRJNA1046507 and listed in Table 1.
